# Screen-detected abnormal ankle brachial index: A risk indicator for future cardiovascular morbidity and mortality in patients with manifest cardiovascular disease

**DOI:** 10.1371/journal.pone.0265050

**Published:** 2022-03-10

**Authors:** Vivianne L. Jagt, Constantijn E. V. B. Hazenberg, Jaap Kapelle, Maarten J. Cramer, Frank L. J. Visseren, Jan Westerink, on behalf of the UCC-SMART Study Group

**Affiliations:** 1 Department of Vascular Medicine, University Medical Center Utrecht, Utrecht, The Netherlands; 2 Department of Vascular Surgery, University Medical Center Utrecht, Utrecht, The Netherlands; 3 Department of Neurology and Neurosurgery, University Medical Center Utrecht, Utrecht, The Netherlands; 4 Department of Cardiology, University Medical Center Utrecht, Utrecht, The Netherlands; Ochsner Clinic Foundation: Ochsner Health System, UNITED STATES

## Abstract

**Objectives:**

The ankle brachial index (ABI) can be used to diagnose peripheral arterial disease (PAD). The clinical relevance of the ABI, especially in patients with known clinically manifest cardiovascular disease (CVD), is unknown. The authors set out to investigate the relationship between a screen-detected ABI and the risk for future cardiovascular morbidity and mortality in patients with clinically manifest CVD.

**Design, materials and methods:**

Patients with clinically manifest CVD were selected from the UCC-SMART cohort (n = 8360) and divided into four groups: normal ABI (0.91–1.39), screen-detected low ABI ≤ 0.9, screen-detected high ABI ≥ 1.4, and patients with known PAD irrespective of their ABI. Adjusted Cox Proportional Hazard Ratios (HRs) for Major Adverse Cardiovascular Events (MACE), Major Adverse Limb Events (MALE), and all-cause mortality were calculated. In addition, stratified analyses for women and men and for the presence of diabetes were performed.

**Results:**

During a median follow-up of 8.3 years (IQR 7.7) 1646 MACE, 601 MALE and 1958 all-cause mortalities were observed. Compared with normal ABI patients, patients with a screen-detected low ABI and patients with manifest PAD had a higher risk of MACE, MALE, and all-cause mortality with HRs of 1.9 (95% CI 1.6–2.2) for MACE, 7.6 (95% CI 5.7–10.1) for MALE, 1.7 (95% CI 1.5–2.0) for mortality and 1.3 (95% CI 1.2–1.5) for MACE, 13.8 (95% CI 11.1–17.1) for MALE, 1.7 (95% CI 1.5–1.9) for mortality, respectively. Screen-detected high ABI did not increase the risk of either MACE or MALE, however, was associated with lower risk of all-cause mortality with a HR of 0.6 (95% CI 0.5–0.9). Stratified analyses for women & men and for diabetes status were comparable for all three outcomes.

**Conclusions:**

In patients with manifest CVD but without PAD, a screen-detected low ABI is a powerful risk indicator for cardiovascular events, limb events, and all-cause mortality.

## Introduction

The ankle brachial index (ABI) is a fast, easy to perform and non-invasive method to diagnose peripheral arterial disease (PAD). The ABI is usually calculated by dividing the systolic blood pressure of the tibial or dorsalis artery by the systolic blood pressure of the brachial artery. Besides its’ use as a diagnostic tool, an abnormal ABI has also been studied as a risk predictor for cardiovascular morbidity and mortality [1–6]. A low ABI is indicative of severe atherosclerosis of the lower extremity arteries and studies, on specific patient populations such as patients with stroke or undergoing CABG, showed that an asymptomatic low ABI score is a strong predictor for future cardiovascular events such as stroke (1.4–5.2), myocardial infarction (2.1–2.4), and mortality (1.6–2.1) [1–6]. High ABI measurements are commonly thought to be indicative of medial arterial calcification (MAC) and/or arterial stiffening and are usually seen in patients with diabetes mellitus (DM), renal failure or the elderly [7–11]. Inconsistent results have been published on the relationship between a high ABI and the risk of future cardiovascular events, with some studies reporting an increased risk [7, 8, 12, 13] and other studies reporting no significant increase in risk [9, 14–16].

Although patients with diverse manifestations of cardiovascular disease (CVD) are at very high risk for future CVD, research into the full range of screen-detected ABI scores with a substantial follow-up period, specifically conducted in this population, is lacking. From a clinical screening standpoint, PAD patients should be reviewed separately. In addition, very few studies on screen-detected ABI scores have focused on female and diabetes patients. This is further emphasized by both the European Society of Cardiology (ESC) and the US Preventive Task Force mandating more research on these two patient populations [17, 18].

The clinical relevance of both low and high screen-detected ABI scores needs to be determined, to predict risk for future cardiovascular events and mortality, but also to predict Major Adverse Limb Events (MALE). This is of great importance as current medical practice focusses on (often complex) prevention of major cardiovascular events, while PAD is easily underdiagnosed and undertreated [19, 20]. Moreover, MALE can lower the quality of life, interfere with physical activity that is necessary for lifestyle changes and impair rehabilitation after CVD events. Therefore, the goal of this study is to determine whether an abnormal low or high screen-detected ABI is a risk indicator for Major Adverse Cardiovascular Events (MACE), MALE and all-cause mortality in patients with manifest CVD. MACE is defined as a composite of stroke, myocardial infarction, terminal heart failure, retinal infarction and/or hemorrhage and CVD death. MALE is defined as a composite of lower limb revascularization (thrombolysis, vascular surgery, or major amputations of the ankle or more proximal). Manifest CVD patients with PAD will be reviewed separately and serve as comparison. In addition, it will be investigated whether the aforementioned associations differ between women and men and between patients with and without diabetes.

## Materials and methods

### Population, baseline measurements and data collection

Patients were selected from the ongoing Utrecht Cardiovascular Cohort–Secondary Manifestations of ARTerial Disease (UCC-SMART, 1996, the Netherlands). For the present study, only patients with clinically manifest CVD at baseline were included (n = 8422). Clinically manifest CVD at baseline was defined as either one of the following or a combination thereof: coronary artery disease (CAD), cerebrovascular disease (CeVD), abdominal aortic aneurysm (AAA) and peripheral arterial disease (PAD). In this prospective cohort, all ABI measurements were taken at baseline and thus a research setting was created that accurately represented screen-detection of abnormal ABI in clinical practice. This study was performed in accordance with the declaration of Helsinki and was approved by the local Utrecht Medical Center ethical committee. Written and oral informed consent was obtained from all patients. Detailed information on the UCC-SMART cohort, vascular disease definitions, and baseline definitions can be found in the Methods section of the Supplemental data [21, 22].

### Formation of study groups according to ABI scores

To interpret the effect of all possible screen-detected ABI scores on future cardiovascular events and interventions, the mixed group of manifest CVD patients were divided into four groups. The first three groups had manifest CVD, but no known PAD. Group 4 consisted of patients with PAD, with or without other manifestations of CVD. From a clinical screening standpoint, patients with known PAD should be reviewed separately and therefore, group 4 was analyzed separately and served as comparison for the three other groups.

**Fig 1:**
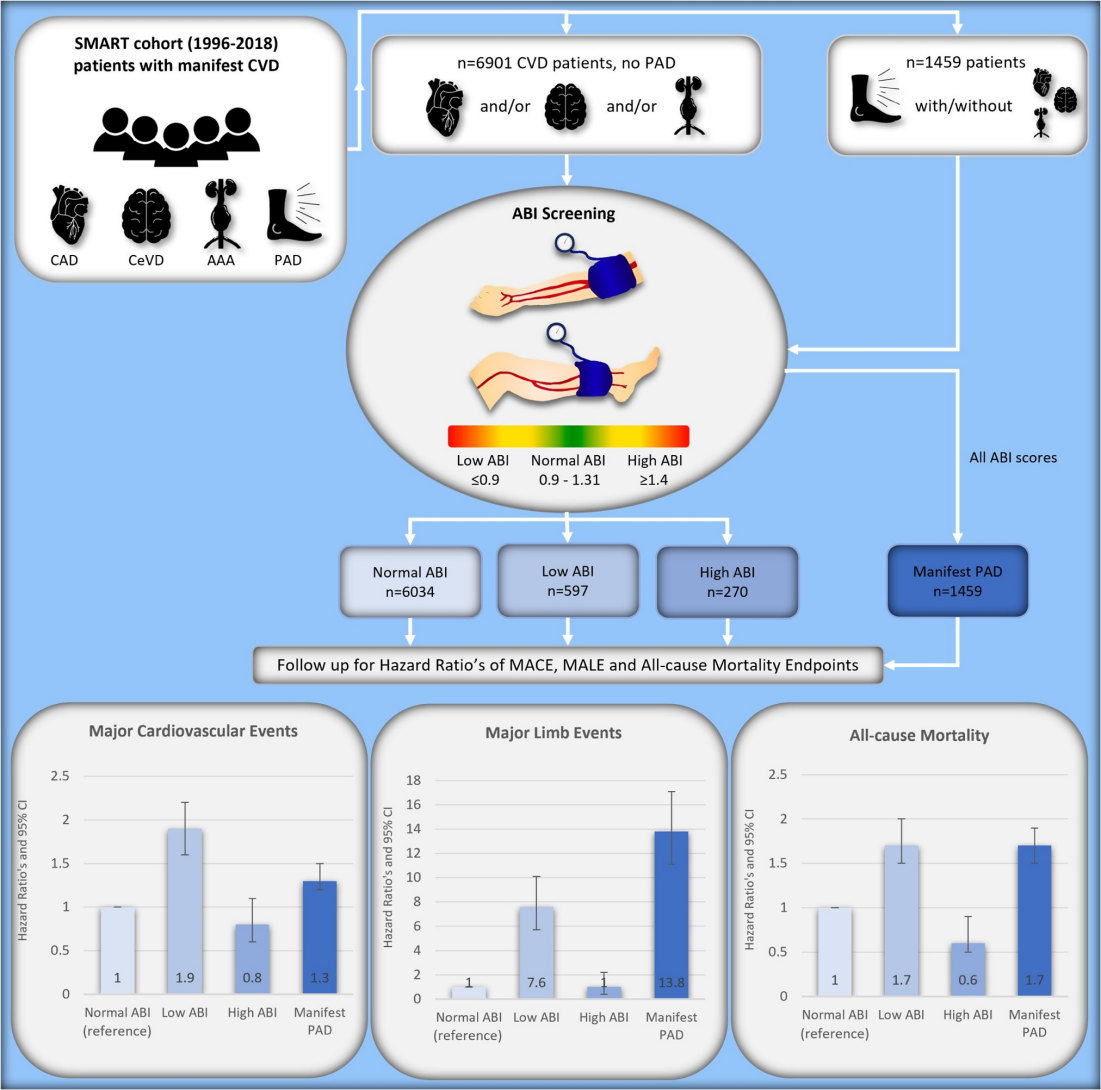
Graphical abstract: Screen-detected ABI scores as risk indicator for future CVD and mortality. Study population, methods, and results for the relationship between screen-detected ABI scores and future cardiovascular events, limb events and all-cause mortality in patients with manifest CVD. MACE was defined a composite of stroke, myocardial infarction, terminal heart failure, retinal infarction and/or hemorrhage and CVD death. MALE was defined as a composite of lower limb revascularization (thrombolysis, vascular surgery, or major amputations of the ankle or more proximal). Graphs show Hazard Ratios adjusted for age, sex, smoking, non-HDL cholesterol, systolic blood pressure, renal function, and diabetes mellitus status. Abbreviations: UCC-SMART = Utrecht Cardiovascular Cohort Second Manifestations of ARTerial disease, CVD = cardiovascular disease, DM = diabetes mellitus, CeVD = cerebrovascular artery disease, CAD = coronary artery disease, AAA = abdominal aortic aneurysm, PAD = peripheral arterial disease, ABI = ankle brachial index, MACE = Major Cardiovascular Adverse Events, MALE = Major Adverse Limb events.

**Fig 2:**
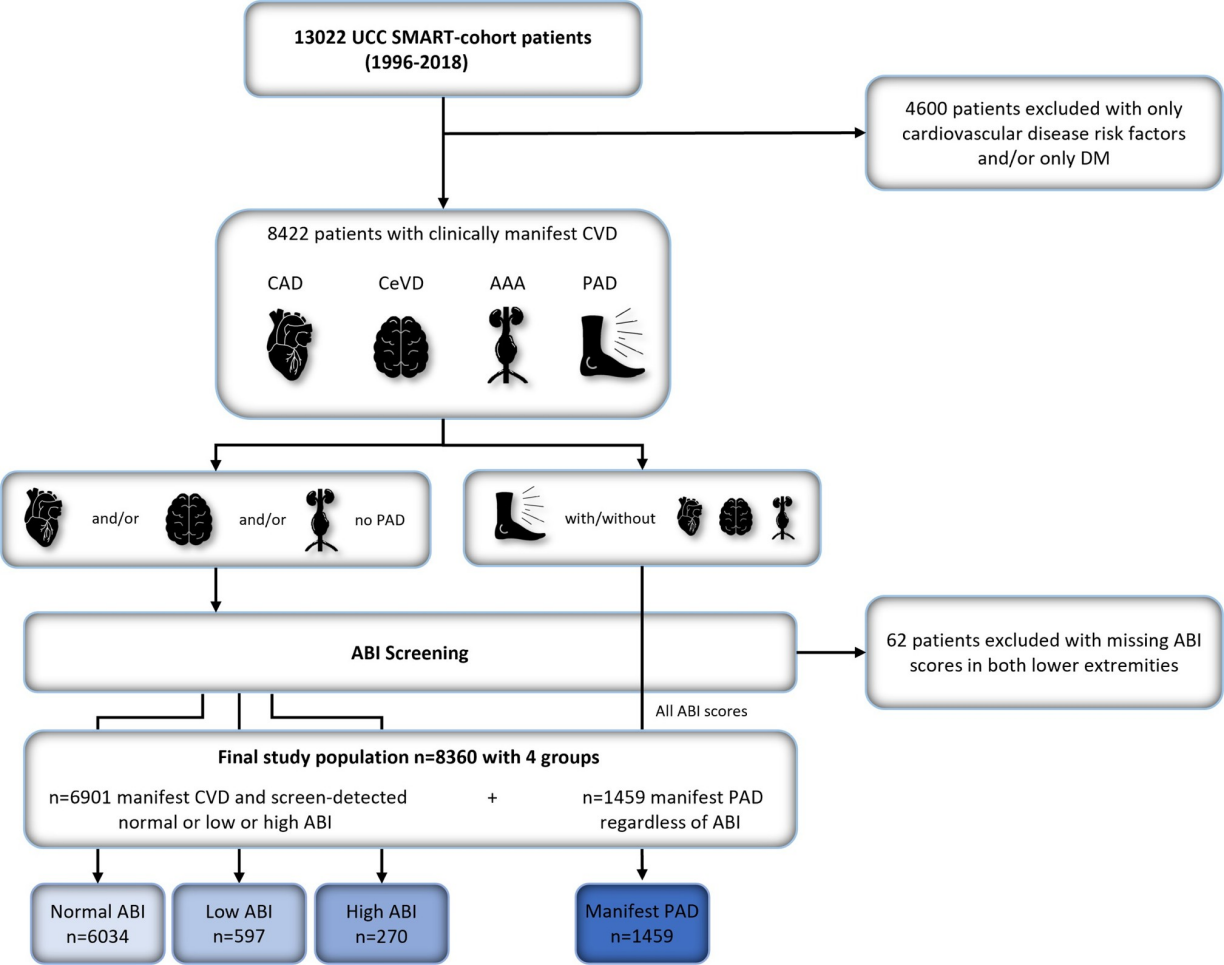
Flowchart of the study population. Abbreviations: UCC-SMART = Utrecht Cardiovascular Cohort Second Manifestations of ARTerial disease, DM = diabetes mellitus, CeVD = cerebrovascular artery disease, CAD = coronary artery disease, AAA = abdominal aortic aneurysm, PAD = peripheral arterial disease, ABI = ankle brachial index.

After ABI screening at baseline, the following groups were formed: Group 1 (n = 6034) consisted of patients with a normal screen-detected ABI score (0.9–1.4 in both legs) with manifest CVD but without PAD, group 2 (n = 597) consisted of patients with a low screen-detected ABI score (≤ 0.9 in at least one leg) with manifest CVD but without PAD, group 3 (n = 270) consisted of patients with a high screen-detected ABI score (ABI ≥ 1.4 in at least one leg) again with manifest CVD but without PAD, and group 4 (n = 1459) consisted of patients with known PAD, regardless of their ABI score at baseline. A visual overview of the study population and the subgroups can be found in the graphical abstract (Fig 1) and Fig 2. Cut-off values for ABI scores were defined according to the European Society of Cardiology (ESC) 2017 guidelines on PAD [17].

Patients with missing ABI in both legs at baseline were excluded (n = 62), resulting in a total study population of 8360 patients. Patients with only one ABI score available at baseline were categorized according to that specific ABI (n = 82). Patients with an ABI score ≤ 0.9 in one leg and an ABI score ≥ 1.4 in the other leg (n = 6) were put in the screen-detected low ABI group, as it was hypothesized that patients with a low ABI were at higher risk of MACE, MALE, and all-cause mortality in comparison to patients with a higher ABI. Average ABI-scores at baseline (Table 1) were calculated by using the mean of the right and left ABI in each patient. ABI range at baseline was calculated using the ABI score of the lower extremity that was used to categorize the patient (Table 1). For patients in group 1 with a normal ABI, the lowest measured ABI score was used. For patients in group 2 and 4, the lowest measured ABI score was used. For patients in group 3, the highest measured ABI score was used.

**Table 1:** Baseline according to ABI and manifest PAD groups.

	Total	CeVD/CAD/AAA			PAD
	n = 8360	n = 6901			n = 1459
		Normal	Screened	Screened	All
		ABI	low ABI	high ABI	ABI
		n = 6034	n = 597	n = 270	
**Demographic data**					
Age	60.1 (10.3)	59.7 (10.3)	64.8 (8.9)	61.6 (9.2)	59.6 (10.4)
Male sex	73.8	74.6	71.9	92.6	67.6
**Lower extremity data**					
Average ABI	1.1 (0.2)	1.2 (0.1)	0.8 (0.1)	1.4 (0.1)	0.9 (0.2)
ABI range	0.0–2.08	0.91–1.39	0.05–0.90	1.40–2.08	0.00–1.90
Symptoms while walking	46.8	33.7	75.9	32.5	91.4
Distance till symptoms					
< 50 m	34.6	36.4	29.9	39.4	32.8
50–500 m	37.2	28.1	47.9	29.6	50.9
500–1000 m	14.4	16.3	14.3	11.3	11.1
>1000 m	13.8	19.2	7.9	19.7	5.3
**History**					
Smoking (current)	30.5	25.0	45.7	9.3	51.0
Packyears	20.0 (19.9)	17.5 (19.0)	30.4 (23.0)	12.6 (15.9)	27.2 (19.8)
Alcohol use (current)	56.2	59.2	47.9	64.4	45.7
Diabetes Mellitus	17.1	15.4	25.1	22.6	20.0
Type 1	0.7	0.6	0.5	0.7	1.2
Type 2	16.4	14.8	24.6	21.9	18.8
Vascular disease					
CAD	61.3	68.7	62.0	77.0	27.8
CVD	29.9	32.6	44.1	25.2	13.6
PAD	17.5	0.0	0.0	0.0	100.0
AAA	8.5	7.8	16.9	4.4	8.3
Vascular disease 1 bed	84.9	91.3	78.6	93.3	59.5
Vascular disease 2 beds	13.1	8.2	19.9	6.7	31.8
Vascular disease ≥3 beds	2.0	0.5	1.5	0.0	8.7
**Physical examination**					
BMI	26.9 (4.0)	27.0 (4.0)	26.6 (4.2)	27.5 (3.8)	26.3 (4.2)
Systolic BP (mmHg)	139 (20.7)	137 (20.0)	145 (22.6)	138 (18.2)	145 (21.6)
Diastolic BP (mmHg)	81 (11.3)	81 (11.3)	80 (11.9)	81 (11.5)	81 (11.3)
**Laboratory data**					
Hba1c % (DM patients)	7.0 (1.2)	6.9 (1.1)	7.1 (1.3)	6.7 (0.9)	7.3 (1.4)
**Medication**					
Insulin	4.5	3.7	7.2	7.8	5.9

Table 1 shows baseline characteristics of patients with clinical manifest cardiovascular disease. Continuous variables are presented as mean with standard deviation (SD), unless otherwise noted. Categorical variables are presented as percentages. PAD at baseline was defined as manifest peripheral arterial disease at baseline and patients with either screen-detected low or high ABI were not classified as PAD patients at baseline. Vascular disease beds consist of CAD, CeVD, PAD and AAA. Extended baseline information on laboratory results and medication use can be found in S1 Table in S1 File. Abbreviations: CeVD = cerebrovascular disease, CAD = coronary artery disease, AAA = abdominal aortic aneurysm, PAD = peripheral arterial disease, ABI = ankle brachial index, BMI = body mass index, SBP = systolic blood pressure, DBP = diastolic blood pressure.

Information on lower extremity symptoms while walking was obtained from questionnaires filled in by study participants at baseline before ABI measurements were taken. Lower extremity symptoms were defined as any feelings of pain, tiredness, heaviness, or tingling feeling in the legs while walking. Both symptomatic (but without known PAD before inclusion) and asymptomatic patients were included, as this accurately represents ABI screening in clinical practice.

### Outcome and follow-up

Biannual questionnaires were filled in by study participants to evaluate the occurrence of new vascular diseases or interventions during follow-up. Patients were followed up from inclusion in the SMART cohort until date of loss to follow-up, death, or the predefined end-date of February 27^th^, 2018 for the present study. All endpoints were reviewed by the UCC-SMART endpoint committee, comprised of physicians from different departments that independently audited all events [21]. For the present study, MACE, MALE and all-cause mortality were studied as outcomes. MACE was defined as a composite of stroke, myocardial infarction, terminal heart failure, retinal infarction and/or hemorrhage and CVD death. MALE was defined as a composite of lower limb revascularization (thrombolysis, vascular surgery, or major amputations of the ankle or more proximal). Only the first MACE and MALE during follow-up were analyzed. MACE and MALE events were followed-up separately.

### Data analysis

Characteristics at baseline were presented for the total study population and for the 4 subgroups separately. Normal distributed variables were represented by means and standard deviations (SD) and non-normal distributed variables were represented by medians and interquartile ranges (IQR). Crude event rates for MACE, MALE and all-cause mortality were calculated per 1000 person years (py). To investigate the relationship between screen-detected ABI scores and the studied outcomes, the Cox Proportional Hazard Regression was used to calculate Hazard Ratios (HRs) for MACE, MALE, and all-cause mortality. The aforementioned 4 ABI/PAD groups were modelled as a categorical variable, with group 1 (normal ABI) as the reference group. The HRs were adjusted for known confounders based on previously reported causal relationships [23–27]. Three different models were constructed adjusting for the following possible confounders: model 1 (age, sex), model 2 (age, sex, smoking, non-HDL cholesterol, systolic blood pressure (SBP)), model 3 (age, sex, smoking, non-HDL cholesterol, SBP, renal function, DM status). Smoking was categorized into current versus non-current (former and never). Age, non-HDL cholesterol, SBP and renal function were all included as continuous variables.

HRs were presented with 95% confidence intervals (95% CI). A p-value ≤ 0.05 was considered statistically significant. As mentioned earlier, due to MAC, the relationship between abnormal ABI-scores and future CVD may differ between DM and non- DM patients and patients with and without renal failure [9, 10, 26, 28–30]. Interaction between ABI and DM and between ABI and eGFR was tested accordingly. Interaction between ABI and sex, smoking and polyvascular disease at baseline was investigated as well. As stated previously, extensive research on the association between screen-detected ABI scores & manifest PAD and MACE, MALE and all-cause mortality in the female and DM population is lacking. Therefore, stratified analyses for women and men and DM and non-DM patients were performed and were corrected according to Models 1,2 and 3 without adjusting for sex or DM, respectively.

Additionally, sensitivity analyses were performed for death as a competing risk. Subdistribution HRs for MACE and MALE (model 3) were computed using a Fine and Gray competing risk regression analysis in R version 3.5.1 (R Foundation for Statistical Computing, Vienna, Austria) and package “cmprsk”.

Selection bias was minimized by imputating missing data in the variables used in the Cox Proportional Hazard Regression with single regression imputation. The original dataset had 0.4% missing data for smoking status (n = 30), 0.2% for SBP (n = 15), 0.6% for non-HDL cholesterol (n = 51) and 0.4% for eGFR (n = 30). The Proportional Hazards Assumption was checked visually by plotting log minus log curves in SPSS and Schoenfeld residuals in R version 3.5.1 (R Foundation for Statistical Computing, Vienna, Austria). All main analyses were performed in SPSS 25.0 for Windows (SPSS Inc., Chicago, IL, USA) except for the competing risk regression and Schoenfeld residuals.

## Results

### Patient characteristics

Out of a total of 8360 (6167 males/2193 females) manifest CVD patients, PAD was present in 1459 patients (17%). The remainder of manifest CVD patients without PAD had a screen-detected normal ABI in 87% (n = 6034), a screen-detected low ABI in 9% (n = 597) and a screen-detected high ABI in 4% (n = 270) as can be seen in Figs 1 and 2. Subdivision of the 4 groups did not remarkably differ between females and males; PAD was present in 22% of female and 16% of male patients. Females with manifest CVD without manifest PAD had a screen-detected normal ABI in 89%, a screen-detected low ABI in 10%, and a screen-detected high ABI in 1%. Males with manifest CVD without manifest PAD had a screen-detected normal ABI in 87%, a screen-detected low ABI in 8%, and a screen-detected high ABI in 5%. Mean age of the total cohort was 60.1 ± 10.3 years (Table 1). Questionnaires filled in prior to ABI measurements revealed that many patients with a screen-detected low ABI (75.9%), did report symptoms while walking, while only 32.5% of patients with a screen-detected high ABI reported symptoms. Symptoms while walking were present in 33.7% of patients with a normal ABI. Detailed information on prevalence of CAD, CeVD, AAA, number of vascular beds affected, DM, smoking at baseline, as well as other risk factors for CVD can be found in Table 1.

### Abnormal screen-detected ABI: Events, event rates and interaction

A total of 1646 MACE, 601 MALE and 1958 all-cause mortalities occurred during a median follow-up of 8.3 (IQR 7.7) years. Overall, patients without manifest PAD, but with screen-detected low ABI had high event rates for MACE (53.35 events/1000 py), MALE (22.67 events/1000 py), and all-cause mortality (55.51 events/1000 py) in comparison with the normal and high ABI patients (Table 2). Across the 4 groups, patients with screen-detected high ABI scores had the lowest event rates for all three outcomes. MALE event rates were highest in the manifest PAD group (34.14 events/1000 py). No clear interaction between ABI and sex, DM, smoking and polyvascular disease at baseline was present when analyzing Hazard Ratio’s for the different groups and outcomes mentioned below.

**Table 2:** Events per 1000 person years.

	Total	CeVD/CAD/AAA			PAD
	n = 8360	n = 6901			n = 1459
		Normal ABI	Screened	Screened	All
			low ABI	high ABI	ABI
		n = 6034	n = 597	n = 270	
**MACE / 1000 PY**	23.57	19.60	53.35	17.00	30.84
**MALE / 1000 PY**	8.47	2.23	22.67	2.26	34.14
**All-cause mortality / 1000 PY**	26.15	20.34	55.51	13.86	41.25
**Sum of all events**	58.19	42.17	131.53	33.12	106.23

Table 2 shows MACE, MALE, and all-cause mortality events per 1000 person years (PY) for patients with screen-detected normal ABI, screen-detected low ABI, screen-detected high ABI, and manifest PAD patients. Abbreviations: CeVD = cerebrovascular disease, CAD = coronary artery disease, AAA = abdominal aortic aneurysm, PAD = peripheral arterial disease, MACE = Major Adverse Cardiovascular Events, MALE = Major Adverse Limb Events.

### Abnormal screen-detected ABI and MACE

**Fig 3:**
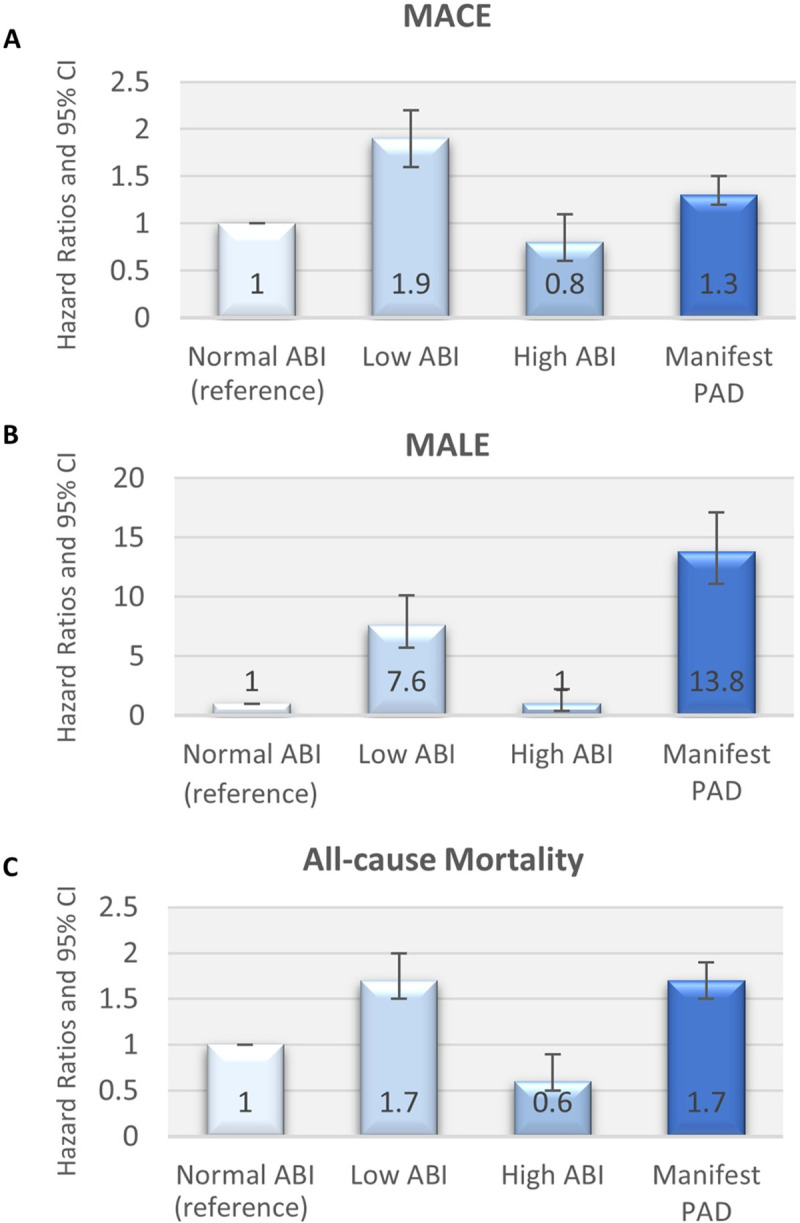
Adjusted Hazard Ratios of screen-detected abnormal ABI & manifest PAD patients for MACE (A), MALE (B), and all-cause mortality (C) according to Model 3. Model 3 was adjusted for age, sex, smoking, non-HDL cholesterol, systolic blood pressure, renal function, and diabetes mellitus status. Abbreviations: ABI = ankle brachial index, PAD = peripheral arterial disease.

Tables 3A–3C, 4A–4C and 5A–5C shows the association between ABI scores and MACE, MALE and all-cause mortality using three different models to correct for confounding, with an increasing number of possible confounders used in each next model. Model 1 was adjusted for age and sex. Model 2 was adjusted for age, sex, smoking, non-HDL cholesterol and systolic blood pressure (SBP). Model 3 was adjusted for age, sex, smoking, non-HDL cholesterol, SBP, renal function, and diabetes mellitus status. As shown in Fig 3A and Table 3A, Cox Proportional HRs for MACE in the screen-detected low ABI and manifest PAD groups remained significantly higher than those of the normal ABI group after adjusting for confounding with Model 3, with HRs of 1.9 (95% CI 1.6–2.2) and 1.3 (95% CI 1.2–1.5) respectively. Screen-detected high ABI was not independently associated with MACE in comparison with the normal ABI group, with a HR of 0.8 (95% CI 0.6–1.1). Subdistribution HRs for MACE were comparable to the Cox Proportional HRs and can be found in the S8 Table in S1 File. Stratified analyses for women and men are presented in Table 3B and 3C. Stratified analyses for diabetes status were comparable and are presented in the S2 and S5 Tables in S1 File.

**Table 3:** The association between screen-detected abnormal ABI scores & manifest PAD and future MACE.

**A**				
**MACE**	**CeVD/CAD/AAA**			**PAD**
*Total population*				
	**Normal ABI**	**Low ABI**	**High ABI**	**All ABI**
	n = 6034	n = 597	n = 270	n = 1459
	987 MACE	220 MACE	42 MACE	397 MACE
		HR (95% CI)	HR (95% CI)	HR (95% CI)
Model 1	Reference	**2.3 (2.0–2.7)**	0.8 (0.6–1.1)	**1.7 (1.5–1.9)**
Model 2	Reference	**2.0 (1.7–2.3)**	0.8 (0.6–1.1)	**1.4 (1.2–1.6)**
Model 3	Reference	**1.9 (1.6–2.2)**	0.8 (0.6–1.1)	**1.3 (1.2–1.5)**
**B**				
**MACE**	**CeVD/CAD/AAA**		**PAD**	
*Women*				
	**Normal ABI**	**Low ABI**	**High ABI**	**All ABI**
	n = 1532	n = 168	n = 20	n = 473
	187 MACE	53 MACE	1 MACE	107 MACE
		HR (95% CI)	HR (95% CI)	HR (95% CI)
Model 1	Reference	**2.4 (1.8–3.3)**	0.4 (0.1–2.7)	**1.7 (1.3–2.1)**
Model 2	Reference	**2.1 (1.6–2.9)**	0.5 (0.0–3.2)	**1.4 (1.1–1.8)**
Model 3	Reference	**1.9 (1.4–2.6)**	0.4 (0.1–3.0)	**1.3 (1.0–1.7)**
**C**				
**MACE**	**CeVD/CAD/AAA**		**PAD**	
*Men*				
	**Normal ABI**	**Low ABI**	**High ABI**	**All ABI**
	n = 4502	n = 429	n = 250	n = 986
	800 MACE	167 MACE	41 MACE	290 MACE
		HR (95% CI)	HR (95% CI)	HR (95% CI)
Model 1	Reference	**2.3 (1.9–2.7)**	0.8 (0.6–1.1)	**1.7 (1.4–1.9)**
Model 2	Reference	**2.0 (1.6–2.3)**	0.9 (0.6–1.2)	**1.4 (1.2–1.6)**
Model 3	Reference	**1.9 (1.6–2.2)**	0.8 (0.6–1.2)	**1.3 (1.2–1.5)**

Table 3A, 3B and 3C show Hazard Ratios for MACE (3A) for the entire study population, MACE (3B) with a stratified analysis for females and MACE (3C) with a stratified analysis for males. All Tables show MACE for normal, low, high ABI and manifest PAD patients separately. Model 1 was adjusted for age and sex. Model 2 was adjusted for age, sex, smoking, non-HDL cholesterol and systolic blood pressure (SBP). Model 3 was adjusted for age, sex, smoking, non-HDL cholesterol, SBP, renal function, and diabetes mellitus status. Abbreviations: CeVD = cerebrovascular artery disease, CAD = coronary artery disease, AAA = abdominal aortic aneurysm, PAD = peripheral arterial disease, ABI = ankle brachial index.

**Table 4:** The association between screen-detected abnormal ABI scores & manifest PAD and future MALE.

**A**				
**MALE**	**CeVD/CAD/AAA**			**PAD**
*Total population*				
	**Normal ABI**	**Low ABI**	**High ABI**	**All ABI**
	n = 6034	n = 597	n = 270	n = 1459
	118 MALE	94 MALE	6 MALE	383 MALE
		HR (95% CI)	HR (95% CI)	HR (95% CI)
Model 1	Reference	**8.9 (6.8–11.7)**	1.0 (0.4–2.2)	**15.8 (12.9–19.4)**
Model 2	Reference	**7.9 (6.0–10.5)**	1.0 (0.4–2.3)	**14.3 (11.5–17.7)**
Model 3	Reference	**7.6 (5.7–10.1)**	1.0 (0.4–2.2)	**13.8 (11.1–17.1)**
**B**				
**MALE**	**CeVD/CAD/AAA**			**PAD**
*Women*				
	**Normal ABI**	**Low ABI**	**High ABI**	**All ABI**
	n = 1532	n = 168	n = 20	n = 473
	23 MALE	24 MALE	0 MALE	110 MALE
		HR (95% CI)	HR (95% CI)	HR (95% CI)
Model 1	Reference	**9.8 (5.5–17.5)**	0.0 (0.0–2.7^163)	**16.5 (10.5–25.9)**
Model 2	Reference	**9.0 (5.0–16.2)**	0.0 (0.0–5.6^172)	**14.7 (9.2–23.5)**
Model 3	Reference	**8.5 (4.7–15.2)**	0.0 (0.0–4.0^169)	**14.7 (9.2–23.5)**
**C**				
**MALE**	**CeVD/CAD/AAA**			**PAD**
*Men*				
	**Normal ABI**	**Low ABI**	**High ABI**	**All ABI**
	n = 4502	n = 429	n = 250	n = 986
	95 MALE	70 MALE	6 MALE	273 MALE
		HR (95% CI)	HR (95% CI)	HR (95% CI)
Model 1	Reference	**8.7 (6.4–11.9)**	1.0 (0.4–2.3)	**15.7 (12.4–19.8)**
Model 2	Reference	**7.6 (5.5–10.5)**	1.0 (0.5–2.4)	**14.2 (11.1–18.2)**
Model 3	Reference	**7.4 (5.4–10.2)**	1.0 (0.4–2.3)	**13.6 (10.6–17.4)**

Table 4A, 4B and 4C show Hazard Ratios for MALE (3D) for the entire study population, MALE (3E) with stratified analysis for females and MALE (3F) with stratified analysis for males. All Tables show MALE for normal, low, high ABI and manifest PAD patients separately. Model 1 was adjusted for age and sex. Model 2 was adjusted for age, sex, smoking, non-HDL cholesterol and systolic blood pressure (SBP). Model 3 was adjusted for age, sex, smoking, non-HDL cholesterol, SBP, renal function, and diabetes mellitus status. Abbreviations: CeVD = cerebrovascular artery disease, CAD = coronary artery disease, AAA = abdominal aortic aneurysm, PAD = peripheral arterial disease, ABI = ankle brachial index.

**Table 5:** The association between screen-detected abnormal ABI scores & manifest PAD and all-cause mortality.

**A**				
**All-cause Mortality**	**CeVD/CAD/AAA**			**PAD**
*Total population*				
	**Normal ABI**	**Low ABI**	**High ABI**	**All ABI**
	n = 6034	n = 597	n = 270	n = 1459
	1093 deceased	259 deceased	37 deceased	569 deceased
		HR (95% CI)	HR (95% CI)	HR (95% CI)
Model 1	Reference	**2.1 (1.8–2.4)**	**0.6 (0.4–0.8)**	**2.1 (1.9–2.4)**
Model 2	Reference	**1.8 (1.6–2.1)**	**0.6 (0.5–0.9)**	**1.8 (1.6–2.0)**
Model 3	Reference	**1.7 (1.5–2.0)**	**0.6 (0.5–0.9)**	**1.7 (1.5–1.9)**
**B**				
**All-cause Mortality**	**CeVD/CAD/AAA**			**PAD**
*Women*				
	**Normal ABI**	**Low ABI**	**High ABI**	**All ABI**
	n = 1532	n = 168	n = 20	n = 473
	207 deceased	59 deceased	2 deceased	167 deceased
		HR (95% CI)	HR (95% CI)	HR (95% CI)
Model 1	Reference	**2.3 (1.7–3.1)**	0.7 (0.2–2.9)	**2.1 (1.7–2.6)**
Model 2	Reference	**2.0 (1.5–2.7)**	0.9 (0.2–3.5)	**1.8 (1.5–2.3)**
Model 3	Reference	**1.8 (1.4–2.5)**	0.8 (0.2–3.1)	**1.8 (1.4–2.2)**
**C**				
**All-cause Mortality**	**CeVD/CAD/AAA**			**PAD**
*Women*				
	**Normal ABI**	**Low ABI**	**High ABI**	**All ABI**
	n = 1532	n = 168	n = 20	n = 473
	207 deceased	59 deceased	2 deceased	167 deceased
		HR (95% CI)	HR (95% CI)	HR (95% CI)
Model 1	Reference	**2.3 (1.7–3.1)**	0.7 (0.2–2.9)	**2.1 (1.7–2.6)**
Model 2	Reference	**2.0 (1.5–2.7)**	0.9 (0.2–3.5)	**1.8 (1.5–2.3)**
Model 3	Reference	**1.8 (1.4–2.5)**	0.8 (0.2–3.1)	**1.8 (1.4–2.2)**

Table 5A, 5B and 5C show Hazard Ratios for all-cause mortality (3G) for the entire study population, all-cause mortality (3H) with stratified analysis for females and all-cause mortality (3I) with stratified analysis for males. All Tables show all-cause mortality for normal, low, high ABI and manifest PAD patients separately. Model 1 was adjusted for age and sex. Model 2 was adjusted for age, sex, smoking, non-HDL cholesterol and systolic blood pressure (SBP). Model 3 was adjusted for age, sex, smoking, non-HDL cholesterol, SBP, renal function, and diabetes mellitus status. Abbreviations: CeVD = cerebrovascular artery disease, CAD = coronary artery disease, AAA = abdominal aortic aneurysm, PAD = peripheral arterial disease, ABI = ankle brachial index.

### Abnormal screen-detected ABI and MALE

Screen-detected low ABI and manifest PAD were independently associated with MALE with HRs of 7.6 (95% CI 5.7–10.1) and 13.8 (95% CI 11.1–17.1) respectively, while screen-detected high ABI was not significantly associated with MALE in comparison with the normal ABI group in Model 3 (Fig 3B, Table 4A). Although statistical significance across the groups did not change, subdistribution HRs for MALE were slightly lower for the screen-detected low ABI and manifest PAD groups compared to the Cox Proportional HRs and can be found in the S9 Table in S1 File. Stratified analyses for women and men are presented in Table 4B and 4C. Stratified analyses for diabetes status were comparable and are presented in the S3 and S6 Tables in S1 File.

### Abnormal screen-detected ABI and all-cause mortality

Screen-detected low ABI and manifest PAD patients had significantly higher HRs of all-cause mortality (1.7, 95% CI 1.5–2.0 and 1.7, 95% CI 1.5–1.9), whereas screen-detected high ABI patients were at a significantly lower hazard (0.6, 95% CI 0.5–0.9) of all-cause mortality in comparison with the normal ABI group in Model 3 (Fig 3C, Table 5A). Stratified analyses for women and men are presented in Table 5B and 5C. Women with a high ABI did not have a significantly higher or lower HR of all-cause mortality, whereas male patients with a high ABI had a significantly lower HR of all-cause mortality. Stratified analyses for diabetes status were comparable and are presented in the S4 and S7 Tables in S1 File.

## Discussion

This study demonstrates that a screen-detected low ABI in patients with manifest CVD but without known PAD, is associated with an increased risk for MACE, MALE, and all-cause mortality. Screen-detected high ABI alone did not increase risk of MACE, MALE, or all-cause mortality in comparison with a normal ABI. These results were similar for women and men and did not change after stratification for the presence of diabetes at baseline either.

The present study investigated both low and high screen-detected ABI scores. This study confirms that the presence of a screen-detected low ABI score independently increases the risk of MACE and mortality in patients that are already at high risk of future CVD. Moreover, the current study included both symptomatic and asymptomatic screen-detected ABI patients and reviewed PAD patients separately, unlike previous studies. This is important as it accurately represents screening in clinical practice. In addition, this study provides novel information by demonstrating that manifest CVD patients (without known PAD) and a low screen-detected ABI are also at high risk for future limb events in comparison with normal ABI patients. The results of low ABI in the current study are in line with previous studies in specific subgroups of CVD patients, which indicated that patients with an asymptomatic abnormal ABI and/or manifest PAD carry a greater risk of stroke, myocardial infarction and mortality [1, 2, 4, 6, 31]. The present study specifically included patients with any form of manifest CVD and combinations thereof, and thus its findings extend to the broad group of patients with CVD.

Screen-detected high ABI in this high-risk population was not associated with an increased risk of cardiovascular and limb events, contrary to some previous studies [7, 8, 12, 13]. Patients in the high ABI group were even at a lower risk of all-cause mortality than patients with a normal ABI. Low event rates were seen in the relatively small high ABI group, which could have led to insufficient power to detect the true effect of high ABI on future CVD events. A recent meta-analysis on this topic showed an overall increased risk of cardiovascular and all-cause mortality in patients with high ABI scores [12]. However, most studies on high ABI scores were conducted in the general population and some studies also included manifest PAD patients in the high ABI groups [12]. As the current study focused on screen-detected ABI in patients with manifest CVD, the added risk associated with an abnormal ABI at screening may be more limited, which has been shown for other markers of subclinical atherosclerosis such as interarm blood pressure differences [32]. This study adds to the published contradictory evidence regarding whether the increased cardiovascular risk in high ABI patients is limited to patients with underlying PAD or not [33, 34]. More research is needed into high ABI scores, the underlying factors and the relationship between high ABI and cardiovascular morbidity and mortality.

Our results did not differ between women and men and between patients with and without DM for all three outcomes. The presence of a high ABI score, such as in patients with DM that are subject to MAC, cannot give conclusive information about the presence of PAD [26, 29]. A suggestion for future screening in patients with high ABI is to use the toe-brachial index in addition to the ABI, as the digital vessels of the toes are usually exempt from MAC [10, 17, 29].

The same effects of abnormal screen-detected ABI scores and manifest PAD in women and men in this cohort could be partially explained by the fact that most women were already postmenopausal, and therefore the protective and immunomodulating effects of estrogen had diminished [35]. Symptoms of PAD in women and men can however largely differ as indicated by previous studies [36]. Prevalence of manifest PAD and screen-detected normal ABI, low ABI and high ABI did not remarkably differ between females and males in this study population. This altogether emphasizes the importance of ABI screening in both men and women with manifest CVD versus solely relying on the presence of PAD symptoms.

### Clinical implications

The addition of ABI measurements to the Framingham Risk Score in the general population has been proven useful and the current study underlines the potential of including ABI measurements to CVD risk scores for manifest CVD patients as well [37]. Another benefit of ABI screening in this high-risk population includes facilitating early treatment of PAD and the observed high cardiovascular risk. This could prevent the large number of cardiovascular and limb events and lead to better quality of life. More attention for PAD and symptoms thereof is needed in clinical practice, as demonstrated by the high percentages of patients with perceived symptoms in this study. Additionally, ABI screening is crucial in detecting PAD and treatment thereof in so-called “masked PAD” patients, which do not experience the classical claudication symptoms due to e.g. neuropathy (DM patients) or not being able to walk the required distance to experience pain (heart disease) [17]. Further research into the possible effects of ABI screening and subsequent treatment based on screening outcomes should be investigated in depth. So far, the only trial conducted on this topic is the AMERICA study, including CAD patients with a mean age of 77 [38]. In this randomized controlled trial, no difference was seen in cardiovascular morbidity and mortality between the multisite arterial disease screening & intensive treatment group and the non-screened & conventional treatment group after a follow-up of 2 years. Although sufficient research on the combination of ABI screening and subsequent treatment is lacking, clinical practitioners can still take advantage of this moment by reiterating the importance of healthy lifestyle choices to the patient in question; such as smoking cessation, adequate daily movement and optimizing nutrition.

### Strengths and limitations

Strengths of the present study are its’ large patient cohort, including a wide variety of manifest CVD patients with a long median follow-up of 8.3 years, and outcome assessment by a clinical endpoint adjudication committee. Extensive analyses were performed with adjustments for confounding and competing risk, along with assessing interaction and providing stratified analyses. Novel insights into the effects of all possible ABI scores and manifest PAD in the female and DM population were provided, as previous studies rarely assessed interaction nor presented stratified analyses. The full range of possible ABI scores was investigated and grouped accordingly to best represent screening in medical practice. This study was also able to provide new information about the relationship between screen-detected abnormal ABI scores in patients with manifest CVD and MALE endpoints. Some limitations of this study need to be addressed. As longitudinal changes in patient medication and treatment were not recorded, it is unknown whether treatment or lifestyle was adjusted after ABI scores at baseline. This quite possibly affected the presented results and could have resulted in underestimation of the true HRs seen in patients with abnormal screen-detected ABI. Furthermore, the number of patients and events in the screen-detected high ABI group was low and therefore the HRs presented for these patients should be interpreted with caution. A toe-brachial index or additional screening in these patients is warranted to diagnose PAD and even more accurately reflect the risk of future CVD.

## Conclusions

In patients with manifest CVD, but without known PAD, screen-detected abnormal ABI scores are prevalent in 13%. Although the ABI score is simple, easy to obtain and non-invasive, a screen-detected low ABI score has proven to be a powerful risk indicator for future cardiovascular events, limb events, and all-cause mortality in patients with manifest CVD. Screen-detected high ABI alone does not seem to increase risk of MACE, MALE, or all-cause mortality. In depth research on the implementation of ABI screening in manifest CVD patients and the effects of consequent treatment after ABI screening is warranted.

## Supporting information

S1 File Supplemental methods, supplemental baseline Table 1, supplemental stratified analyses DM Tables 2–7, supplemental subdistribution analyses Tables 8–9, and supplemental references. (DOCX)

## Non-standard abbreviations

ABI: Ankle Brachial Index
CVD: Cardiovascular Disease
CeVD: Cerebrovascular Disease
CAD: Coronary Artery Disease
PAD: Peripheral Arterial Disease
95% CI: 95% Confidence Interval
HR: Hazard Ratio
MAC: Medial Arterial Calcification
