# Supplementary material for: Screen-detected abnormal ankle brachial index: A risk indicator for future cardiovascular morbidity and mortality in patients with manifest cardiovascular disease
Source: PLoS One. 2022 Mar 10;17(3):e0265050. doi: 10.1371/journal.pone.0265050 (PMC8912207; doi:10.1371/journal.pone.0265050)
Supplement: S1 File — (DOCX) [file pone.0265050.s001.docx]

**Supplemental material**

**Screen-detected abnormal ankle brachial index: A risk indicator for future cardiovascular morbidity and mortality in patients with manifest cardiovascular disease**

Vivianne L. Jagt^1^, Constantijn E.V.B. Hazenberg^2^, Jaap Kapelle^3^, Maarten J. Cramer^4^, Frank L.J. Visseren^1^, Jan Westerink^1*^, on behalf of the UCC-SMART Study Group^^^.

1. Department of Vascular Medicine, University Medical Center Utrecht, Utrecht, The Netherlands.

2. Department of Vascular Surgery, University Medical Center Utrecht, Utrecht, The Netherlands.

3. Department of Neurology and Neurosurgery, University Medical Center Utrecht, Utrecht, The Netherlands.

4. Department of Cardiology, University Medical Center Utrecht, Utrecht, The Netherlands.

^ All members of the UCC-SMART Study group are listed in the acknowledgement section.

Word count supplemental data (including title page, Tables, and legends): 2151.

Supplemental data 9 Tables.

*Corresponding author

Email: [J.Westerink-3@umcutrecht.nl](mailto:J.Westerink-3@umcutrecht.nl) (JW)

**Supplemental methods**

**Population**

Patients were selected from the ongoing Utrecht Cardiovascular Cohort – Secondary Manifestations of ARTerial Disease (UCC-SMART, 1996, the Netherlands). The SMART study is a prospective, single-center cohort study which includes patients aged 18-79 presenting with manifest CVD or requiring treatment of cardiovascular risk factors. Exclusion criteria are presence of terminal malignancy, not being able to perform daily activities independently, insufficient fluency in the Dutch language or referral back to the referring clinician after one visit. Comprehensive information on the UCC-SMART cohort has been described previously [1].

For the present study, only patients with clinically manifest CVD at baseline were included (n=8422). Patients with only risk factors for CVD and/or only DM were excluded. Clinically manifest CVD at baseline was defined as either one of the following or a combination thereof: cerebrovascular disease (CeVD), coronary artery disease (CAD), abdominal aortic aneurysm (AAA) and peripheral arterial disease (PAD). CeVD was defined as clinical diagnosis of a transient ischemic attack, transient ischemic stroke, hemorrhagic stroke, or history of carotid artery (endovascular) surgery. CAD was defined as clinical diagnosis of angina pectoris, myocardial infarction, cardiac arrest, history of coronary bypass surgery or coronary angioplasty. AAA was defined as an abdominal aortic aneurysm ≥ 3 cm at baseline or history of abdominal aortic surgery. PAD was defined as documented obstruction of the distal arteries (either resting ABI ≤0.90 or post-exercise decrease of ≥ 20% in at least one ABI) together with symptoms corresponding to PAD, a history of percutaneous transluminal angioplasty, bypass surgery, endarterectomy (or a combination of these) or prior amputation of the extremity due to ischemia.

**Baseline measurements and data collection**

Information about medical history, clinical and laboratory measurements, medication, lifestyle and other patient characteristics was gathered at baseline in our tertiary vascular referral center using an elaborate standardized protocol described elsewhere [1]. Diabetes mellitus (DM) at baseline was defined as self-reported DM type 1 or 2, the presence of fasting plasma glucose ≥ 7 mmol/L at baseline, use of oral anti-diabetic drugs, use of insulin or initiating treatment with oral anti-diabetic drugs/insulin within the first year after inclusion. Vascular disease beds affected at baseline were categorized into 1 vascular disease, 2 vascular disease beds or ≥ 3 vascular disease beds. CAD, CVD, AAA and/or PAD at baseline were used to calculate the number of affected vascular disease beds in the above said categories.

Blood pressure (BP) was measured twice in sitting position in both the right and left upper arm and was represented by the mean of the two measurements in the arm with the highest BP measurement. Renal function was estimated using the CKD-EPI formula and presented as estimated Glomerular Filtration Rate (eGFR). All cholesterol laboratory values were measured using fasting venous blood samples. LDL-cholesterol was calculated using the Friedewald formula. Non-HDL cholesterol was calculated by subtracting HDL-cholesterol from total cholesterol. Micro and macro-albuminuria were calculated by using immunoturbidimetric assays on urine samples and cut-off values were defined according to the KDIGO guideline [2].

Lipid lowering medication was defined as any of the following: the use of statins, fibric acid derivatives, bile acid sequestrants, cholesterol absorption inhibitors, and/or the use of other types of lipid lowering medication. Blood pressure (BP) lowering medication was defined as any of the following: the use of beta-receptor blocking agents, diuretics, loop diuretics, thiazide diuretics, potassium-sparing diuretics, aldosterone receptor antagonists, ACE inhibitors, calcium antagonists, selective alpha-1 adrenoreceptor antagonists, centrally acting antihypertensive agents, angiotensin II receptor antagonists, direct-acting vasodilators, and/or a combination of the aforementioned. Glucose lowering medication was defined as the use of any glucose lowering medication besides insulin. Oral anticoagulation was defined as the use of oral vitamin K inhibitors. Thrombocyte aggregation inhibitors were defined as the use of acetylsalicylic acid, carbasalate calcium, P2Y12 inhibitors, Dipyridamol, Persantin, and/or a combination of the aforementioned. Medication use, smoking habits and alcohol use were self-reported. Alcohol use was defined as any use of alcoholic beverages and categorized into current, past, and never.

**ABI measurements**

All ABI measurements were conducted at baseline by experienced professionals in the UMC Utrecht vascular lab. The systolic blood pressure (SBP) of the Posterior Tibial and Dorsalis Pedis arteries was measured bilaterally using an 8MHz Vasoguard Doppler probe. The SBP of the brachial artery was measured twice in sitting position in both the right and left upper arm. The mean of the two measurements in the arm in which the highest BP was measured was used as the denominator in both the right and left ABI formula. The right and left ABI were subsequently calculated using the formulas described below.

$$ABI Right=\frac{Highest SBP of right ankle (either Tibial or Dorsalis)}{highest SBP brachial average}$$

$$ABI Left=\frac{Highest SBP of left ankle (either Tibial or Dorsalis)}{highest SBP brachial average}$$

Changing treatment or advising lifestyle changes after ABI measurements at baseline was at the discretion of the treating specialist or general practitioner. Information on longitudinal changes of baseline medication and lifestyle was not recorded.

**Supplemental Tables**

**S1 Table. Baseline information on laboratory data and medication use according to ABI and manifest PAD groups.**

|  | **Total**  n=8360 | **CeVD/CAD/AAA**  n = 6901 | | | **PAD**  n=1459 |
| --- | --- | --- | --- | --- | --- |
|  |  | **Normal**  **ABI**  n = 6034 | **Screened**  **low ABI**  n = 597 | **Screened**  **high ABI**  n = 270 | **All**  **ABI** |
| **Laboratory data** |  |  |  |  |  |
| TC (mmol/L) | 4.8 (1.2) | 4.7 (1.2) | 4.9 (1.2) | 4.5 (1.0) | 5.3 (1.2) |
| LDL (mmol/L) | 2.8 (1.0) | 2.7 (1.0) | 2.9 (1.0) | 2.6 (0.9) | 3.2 (1.1) |
| HDL (mmol/L) | 1.2 (0.4) | 1.2 (0.4) | 1.2 (0.3) | 1.2 (0.3) | 1.2 (0.4) |
| Triglycerides (mmol/L)  median + IQR | 1.4 (1.0) | 1.3 (0.9) | 1.5 (1.1) | 1.4 (0.8) | 1.6 (1.2) |
| non-HDL-c (mmol/L) | 3.6 (1.2) | 3.5 (1.2) | 3.7 (1.2) | 3.3 (1.0) | 4.1 (1.3) |
| eGFR (ml/min/1.73 m^2^) | 78 (17.9) | 78 (17.3) | 71 (19.5) | 77 (15.6) | 77 (19.6) |
| Albuminuria  Macro  Micro | 1.7  11.4 | 1.2  9.1 | 3.8  22.8 | 1.6  9.8 | 3.1  16.6 |
| CRP mg/L median + IQR | 2.0 (3.4) | 1.8 (2.9) | 3.2 (4.6) | 1.6 (2.8) | 3.1 (4.8) |
| **Medication** |  |  |  |  |  |
| Lipid lowering | 69.0 | 72.8 | 72.4 | 80.0 | 50.0 |
| BP lowering | 75.1 | 79.0 | 80.2 | 82.2 | 55.4 |
| Glucose lowering | 10.9 | 10.2 | 14.9 | 15.2 | 11.1 |
| OAC | 10.8 | 9.6 | 13.7 | 17.0 | 13.8 |
| TAI | 77.1 | 81.3 | 79.4 | 78.9 | 58.5 |

S1 Table shows baseline characteristics of patients with clinical manifest cardiovascular disease. Continuous variables are presented as mean with standard deviation (SD), unless otherwise noted. Categorical variables are presented as percentages. PAD at baseline was defined as manifest peripheral arterial disease at baseline and patients with either screen-detected low or high ABI were not classified as PAD patients at baseline. Abbreviations: CeVD = cerebrovascular disease, CAD = coronary artery disease, AAA = abdominal aortic aneurysm, PAD = peripheral arterial disease, ABI = ankle brachial index, TC = total cholesterol, LDL = low density lipoprotein, HDL = high density lipoprotein, non-HDL-c = non-high density lipoprotein cholesterol, eGFR = estimated glomerular filtration rate, OAC = oral anti-coagulation, TAI = thrombocyte aggregation inhibitor.

**S2 Table.** **Stratified analyses DM patients: MACE.**

| **MACE** | **CeVD/CAD/AAA** | | | **PAD** |
| --- | --- | --- | --- | --- |
|  | **Normal ABI**  n = 930  214 MACE | **Low ABI**  n = 150  51 MACE  HR (95% CI) | **High ABI**  n = 61  16 MACE  HR (95% CI) | **All ABI**  n = 292  107 MACE  HR (95% CI) |
| Model 1 | Reference | **1.6 (1.2-2.2)** | 1.2 (0.7-1.9) | **1.6 (1.3-2.0)** |
| Model 2 | Reference | **1.4 (1.0-2.0)** | 1.2 (0.7-2.1) | **1.4 (1.1-1.7)** |
| Model 3 | Reference | **1.4 (1.0-1.9)** | 1.1 (0.7-1.9) | **1.4 (1.1-1.7)** |

Abbreviations: CeVD = cerebrovascular disease, CAD = coronary artery disease, AAA = abdominal aortic aneurysm, PAD = peripheral arterial disease, MACE = Major Adverse Cardiovascular Events, MALE = Major Adverse Limb Events.

**S3 Table. Stratified analyses DM patients: MALE.**

| **MALE** | **CeVD/CAD/AAA** | | | **PAD** |
| --- | --- | --- | --- | --- |
|  | **Normal ABI**  n = 930  38 MALE | **Low ABI**  n = 150  25 MALE  HR (95% CI) | **High ABI**  n = 61  5 MALE  HR (95% CI) | **All ABI**  n = 292  100 MALE  HR (95% CI) |
| Model 1 | Reference | **5.0 (3.0-8.4)** | 1.9 (0.8-4.9) | **11.0 (7.6-16.0)** |
| Model 2 | Reference | **5.1 (3.0-8.5)** | 1.9 (0.8-4.9) | **10.7 (7.3-15.7)** |
| Model 3 | Reference | **5.1 (3.0-8.5)** | 1.9 (0.8-4.9) | **10.7 (7.3-15.7)** |

Abbreviations: CeVD = cerebrovascular disease, CAD = coronary artery disease, AAA = abdominal aortic aneurysm, PAD = peripheral arterial disease, MACE = Major Adverse Cardiovascular Events, MALE = Major Adverse Limb Events.

**S4 Table. Stratified analyses DM patients: all-cause mortality.**

| **All-cause mortality** | **CeVD/CAD/AAA** | | | **PAD** |
| --- | --- | --- | --- | --- |
|  | **Normal ABI**  n = 930  235 deceased | **Low ABI**  n = 150  66 deceased  HR (95% CI) | **High ABI**  n = 61  16 deceased  HR (95% CI) | **All ABI**  n = 292  143 deceased  HR (95% CI) |
| Model 1 | Reference | **1.9 (1.5-2.6)** | 1.0 (0.6-1.7) | **1.9 (1.6-2.4)** |
| Model 2 | Reference | **1.7 (1.3-2.2)** | 1.1 (0.6-1.8) | **1.7 (1.3-2.1)** |
| Model 3 | Reference | **1.7 (1.3-2.2)** | 1.0 (0.6-1.7) | **1.7 (1.3-2.1)** |

Abbreviations: CeVD = cerebrovascular disease, CAD = coronary artery disease, AAA = abdominal aortic aneurysm, PAD = peripheral arterial disease, MACE = Major Adverse Cardiovascular Events, MALE = Major Adverse Limb Events.

**S5 Table. Stratified analyses non-DM patients: MACE.**

| **MACE** | **CeVD/CAD/AAA** | | | **PAD** |
| --- | --- | --- | --- | --- |
|  | **Normal ABI**  n = 5104  773 MACE | **Low ABI**  n = 447  169 MACE  HR (95% CI) | **High ABI**  n = 209  26 MACE  HR (95% CI) | **All ABI**  n = 1167  290 MACE  HR (95% CI) |
| Model 1 | Reference | **2.5 (2.1-3.0)** | 0.6 (0.4-0.9) | **1.6 (1.4-1.9)** |
| Model 2 | Reference | **2.1 (1.8-2.6)** | 0.7 (0.5-1.0) | **1.4 (1.2-1.7)** |
| Model 3 | Reference | **2.1 (1.7-2.5)** | 0.7 (0.5-1.0) | **1.3 (1.2-1.5)** |

Abbreviations: CeVD = cerebrovascular disease, CAD = coronary artery disease, AAA = abdominal aortic aneurysm, PAD = peripheral arterial disease, MACE = Major Adverse Cardiovascular Events, MALE = Major Adverse Limb Events.

**S6 Table. Stratified analyses non-DM patients: MALE.**

| **MALE** | **CeVD/CAD/AAA** | | | **PAD** |
| --- | --- | --- | --- | --- |
|  | **Normal ABI**  n = 5104  80 MALE | **Low ABI**  n = 447  69 MALE  HR (95% CI) | **High ABI**  n = 209  1 MALE  HR (95% CI) | **All ABI**  n = 1167  283 MALE  HR (95% CI) |
| Model 1 | Reference | **10.7 (7.8-14.9)** | 0.3 (0.0-1.8) | **17.7 (13.8-22.8)** |
| Model 2 | Reference | **9.0 (6.5-12.6)** | 0.3 (0.0-1.9) | **15.3 (11.8-19.9)** |
| Model 3 | Reference | **9.1 (6.5-12.7)** | 0.3 (0.0-1.9) | **15.3 (11.8-19.9)** |

Abbreviations: CeVD = cerebrovascular disease, CAD = coronary artery disease, AAA = abdominal aortic aneurysm, PAD = peripheral arterial disease, MACE = Major Adverse Cardiovascular Events, MALE = Major Adverse Limb Events.

**S7 Table.** **Stratified analyses non-DM patients: all-cause mortality.**

| **All-cause mortality** | **CeVD/CAD/AAA** | | | **PAD** |
| --- | --- | --- | --- | --- |
|  | **Normal ABI**  n = 5104  858 deceased | **Low ABI**  n = 447  193 deceased  HR (95% CI) | **High ABI**  n = 209  21 deceased  HR (95% CI) | **All ABI**  n = 1167  426 deceased  HR (95% CI) |
| Model 1 | Reference | **2.1 (1.8-2.5)** | **0.4 (0.3-0.7)** | **2.2 (1.9-2.5)** |
| Model 2 | Reference | **1.8 (1.5-2.1)** | **0.5 (0.3-0.8)** | **1.8 (1.6-2.0)** |
| Model 3 | Reference | **1.7 (1.5-2.0)** | **0.5 (0.3-0.8)** | **1.8 (1.5-2.0)** |

Abbreviations: CeVD = cerebrovascular disease, CAD = coronary artery disease, AAA = abdominal aortic aneurysm, PAD = peripheral arterial disease, MACE = Major Adverse Cardiovascular Events, MALE = Major Adverse Limb Events.

**S8 Table. Subdistribution HRs for MACE.**

| **MACE** | **CeVD/CAD/AAA** | | | **PAD** |
| --- | --- | --- | --- | --- |
|  | **Normal ABI**  n = 6034  987 MACE | **Low ABI**  n = 597  220 MACE  HR (95% CI) | **High ABI**  n = 270  42 MACE  HR (95% CI) | **All ABI**  n = 1459  397 MACE  HR (95% CI) |
| Model 3 | Reference | **1.8 (1.6-2.1)** | 0.9 (0.6-1.2) | **1.3 (1.1-1.4)** |

Subdistribution HRs for MACE according to competing risk analysis for death. Abbreviations: CeVD = cerebrovascular disease, CAD = coronary artery disease, AAA = abdominal aortic aneurysm, PAD = peripheral arterial disease, MACE = Major Adverse Cardiovascular Events, MALE = Major Adverse Limb Events.

**S9 Table. Subdistribution HRs for MALE.**

| **MALE** | **CeVD/CAD/AAA** | | | **PAD** |
| --- | --- | --- | --- | --- |
|  | **Normal ABI**  n = 6034  118 MALE | **Low ABI**  n = 597  94 MALE  HR (95% CI) | **High ABI**  n = 270  6 MALE  HR (95% CI) | **All ABI**  n = 1459  383 MALE  HR (95% CI) |
| Model 3 | Reference | **7.0 (5.3-9.4)** | 1.0 (0.5-2.3) | **12.7 (10.1-15.9)** |

Subdistribution HRs for MALE according to competing risk analysis for death. Abbreviations: CeVD = cerebrovascular disease, CAD = coronary artery disease, AAA = abdominal aortic aneurysm, PAD = peripheral arterial disease, MACE = Major Adverse Cardiovascular Events, MALE = Major Adverse Limb Events.

**Supplemental references**

1. Simons PCG, Algra A, Van De Laak MF, Grobbee DE, Van Der Graaf Y. Second manifestations of ARTerial disease (SMART) study: Rationale and design. Eur J Epidemiol. 1999; 15: 773–781.

2. International Society of Nephrology. KDIGO 2012 Clinical Practice Guideline for the Evaluation and Management of Chronic Kidney Disease. Kidney Int Suppl; 3. Epub ahead of print 2013. doi: 10.1038/kisup.2012.73.
